# Diagnosing Perioperative Cardiovascular Risks in Noncardiac Surgery Patients

**DOI:** 10.1155/2019/6097375

**Published:** 2019-08-25

**Authors:** Panpan Li, Ying Lei, Qiaomei Li, Thangavel Lakshmipriya, Subash C. B. Gopinath, Xinwen Gong

**Affiliations:** ^1^Department of Encephalopathy, Ankang Traditional Chinese Medicine Hospital, No. 47, Bashan East Road, Hanbin District, Ankang City, Shaanxi Province 725000, China; ^2^Department of Functional (ECG Room), Ankang Traditional Chinese Medicine Hospital, No. 47, Bashan East Road, Hanbin District, Ankang City, Shaanxi Province 725000, China; ^3^Operating Room, Ankang Traditional Chinese Medicine Hospital, No. 47, Bashan East Road, Hanbin District, Ankang City, Shaanxi Province 725000, China; ^4^Institute of Nano Electronic Engineering, Universiti Malaysia Perlis, 01000 Kangar, Perlis, Malaysia; ^5^School of Bioprocess Engineering, Universiti Malaysia Perlis, 02600 Arau, Perlis, Malaysia; ^6^Department of Cardiology, Ankang Traditional Chinese Medicine Hospital, No. 47, Bashan East Road, Hanbin District, Ankang City, Shaanxi Province 725000, China

## Abstract

Every year, over 200 million adults are undergoing noncardiac surgery. These noncardiac surgery patients may face the risk of cardiac mortality and morbidity during the perioperative and recovery periods. Around ten million patients who underwent noncardiac surgery experience cardiac complications within the first 30 days of the postoperative period; the complications are myocardial infarction, cardiac death, and cardiac arrest. This cardiovascular risk is mostly faced by the patients having cerebrovascular or cardiac disease and the patients with the age greater than 50 years. Monitoring and treating cardiac diseases with a suitable biomarker during the perioperative period is necessary for the early recovery of noncardiac surgery patients. This review discussed the risk factors and the key guidelines to avoid the cardiovascular risks during the perioperative period of noncardiac surgery patients. In addition, the biomarkers and identification strategies for cardiac diseases are discussed.

## 1. Introduction

In the past, over 50 million surgeries have been performed every year in America; among them, 1.4–3.9% of patients are facing the complications by cardiac issues [1]. Other surgery cases in the rest of the world are also showing the similar problems, and the mandatory urging attempts have been made to overcome the above issues. Among these, analyzing the cardiovascular risks during the perioperative period of noncardiac surgery patients is the common clinical practice to take care of the associated cardiovascular problems by the anesthesiologist, medical consultant, and surgeon [2]. This practice involves managing and detecting cardiovascular diseases and predicting the long and short periods with cardiovascular risks [3]. In particular, the analyses are necessary for the patients with the age above 50 years and the patients already having the cardiovascular problems owing to the pulmonary edema, acute myocardial infarction, and primary cardiac death [4]. So far, ischemic heart disease for noncardiac surgery patients during the preoperative evaluation is the most common cardiac issue. The goal of analyzing preoperative cardiovascular risk management is to develop a patient's good health. In this review, the authors discussed the possible reasons of cardiovascular risks during noncardiac surgery and assessed the clinical issues during the preoperative period, biomarkers for preoperative analyses, and guidelines and recommendations for the preoperative cardiovascular risk assessment.

## 2. Reasons for Increasing Risks and Causes of Risks Associated with Surgery

The cardiovascular risks are much higher in the patients having cardiovascular-related problems. These risks are depending on various factors including the patients with cardiovascular history, fluid exchanges, and the type of anesthesia [5, 6]. It has been found that with a patient there were postoperative cardiovascular complications such as atrial fibrillation. In particular, the risk is connected with coronary artery disease [7]; in addition, obesity increases cardiovascular risks. The patients with obesity have increased risk of an adverse cardiovascular problem during the period of noncardiac surgery [8].

Age is also considered as one of the important factors for cardiovascular risks during the time of noncardiac surgery. Patients aged above 55 years with cardiovascular disease/cerebrovascular disease and diabetes will have more risk of different cardiovascular-related problems such as heart failure, valvular heart disease, myocardial infarction, and pulmonary vascular disease. The patients with the age above 62 years have the enhanced risk of perioperative stroke. For the age above 65 years, there was an evidenced report showing the risk of acute ischemic stroke while undergoing noncardiac surgery. Obviously, patients over the age of 70 years are facing lots of postoperative complications [9].

Heart failure is one of the major perioperative risks during noncardiac surgery [10]. Hammill et al. [10] concluded that patients having coronary artery disease and heart failure are facing the highest risk during noncardiac surgery. Patients with distended jugular veins or third sound at the preoperative examination show the high risk of postoperative pulmonary edema. The risk is higher in the patients who have the left ventricular dysfunction, asymptomatic cardiac stress, ischemic heart disease and arrhythmias, and valvular heart disease [11]. In addition, also the patients with the record of congestive heart failure during the chest roentgenogram have the risk of perioperative pulmonary edema [12–14]. It was found that the patients with heart failure have the minor ambulatory. Unfortunately, a death rate of 4.8% is with the nonischemic heart failure cases compared to 0.8% of the coronary artery disease patients [15].

## 3. Assessing Clinical Data for Perioperative Evaluation

**Figure 1:**
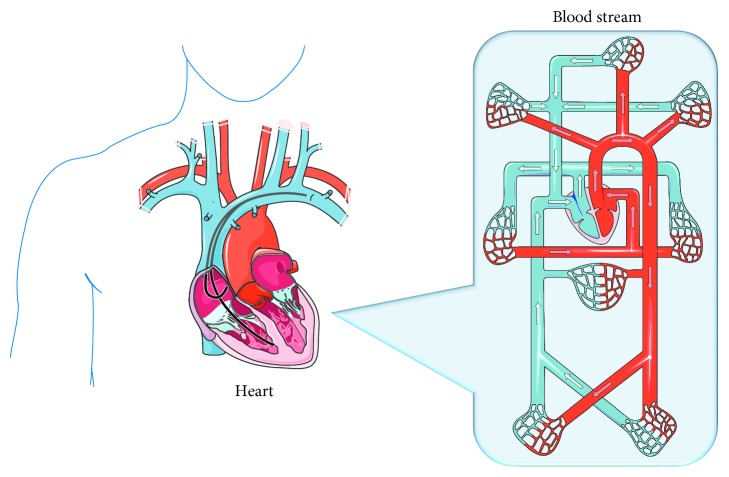
Overview of the human heart and the blood streaming system.

**Figure 2:**
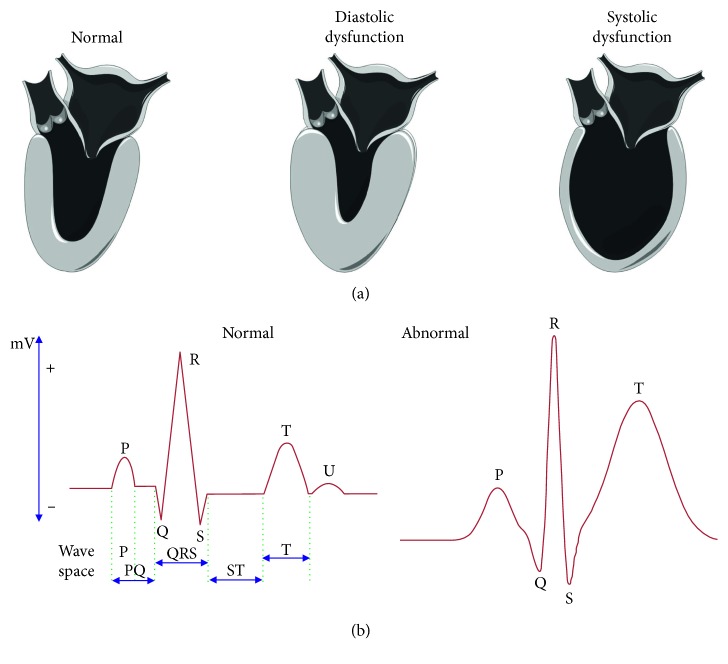
System correlation with the heart. (a) Normal, diastolic, and systolic dysfunction of the heart. (b) Normal and abnormal electrocardiograms.

The patient's history with the physical examination reveals the possible risk factors for pulmonary, cardiac, and infectious diseases, and the analysis on the functional capacity of the patients is considered as the perioperative evaluation [16]. The patient's medical record and clinical data are investigated to monitor the basic function of the heart. The normal healthy man has a properly functioning heart with a good bloodstream (Figure 1). Basic laboratory tests including chest X-ray, body mass index, blood test, and electrocardiography (ECG) have been recorded before the four weeks of the surgery. The assessment on diastolic and systolic dysfunctions is the supporting measurement for the above tests (Figure 2(a)). ECG is one of the very common practices to monitor cardiac failure as the preliminary test (Figure 2(b)). In addition, the functional capacity of the patients has been analyzed by a spectrum with the daily activities.

## 4. Biomarkers for Perioperative Evaluation

Even though the risk factors for cardiovascular disease have been found to be decreased recent years, most of the deaths (∼50%) are caused due to the cardiovascular complaints in the patients already having the history of cardiovascular problems [17]. To overcome this issue, it is mandatory to use the suitable cardiac-specific biomarkers towards the diagnosis. Analysis on these biomarkers helps to reveal the problems associated with the heart muscle, myocardial stress, apoptosis, and neurohormonal pathways. The predominant and common biomarkers are CK-MB (creatine kinase-MB) isoenzyme, CK (creatine kinase), AST (aspartate aminotransferase), HDBH (hydroxyl butyrate dehydrogenase), LDH (lactate dehydrogenase), TnT (troponin T), TnI (troponin I), and myoglobin. Developing a novel biomarker is mandatory to avoid the cardiovascular risks during the perioperative period. Karp [18] has analyzed the biomarkers with 2054 noncardiac surgery patients; it has been found that N-terminal pro-B-type natriuretic peptide (NT-proBNP) and C-reactive protein (CRP) were independent and strong biomarkers for the perioperative cardiovascular risk event. It was found that using NT-BNP, it is possible to predict the major cardiovascular risks or deaths in the patients having heart failure and coronary artery disease [19]. With CRP marker-associated perioperative cardiovascular risks, huge cohort of patients are undergoing a major elective noncardiac surgery [20]. An elevated level of troponin has also been found to be an indication of cardiovascular risk [21]. Another research has found that the increasing level of troponin was noticed in patients who are undergoing leg amputation with chronic peripheral arterial vascular disease [22] and in the patients having the history of chronic critical limb ischaemia [23]. In addition, hsTnT (high-sensitive troponin), hFABP (heart-type fatty acid-binding protein), miRNA (microRNA), and MR-PAMAP (midregional fragment of proadrenomedullin) were also used as the biomarkers for cardiovascular risk monitoring [24].

## 5. Diagnosing Cardiovascular Risk-Associated Biomarkers by Biosensors

**Figure 3:**
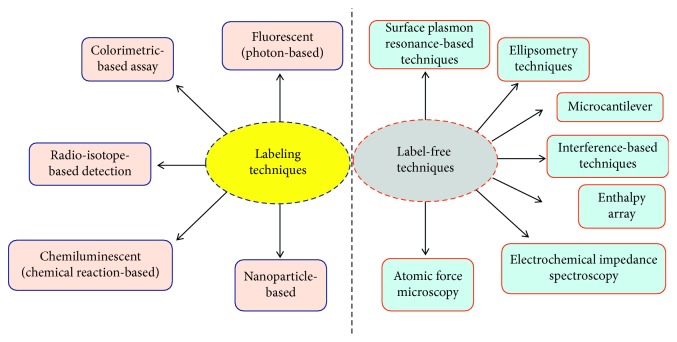
Strategies with biosensors. Different sensing systems with labelling and label-free strategies are displayed.

**Figure 4:**
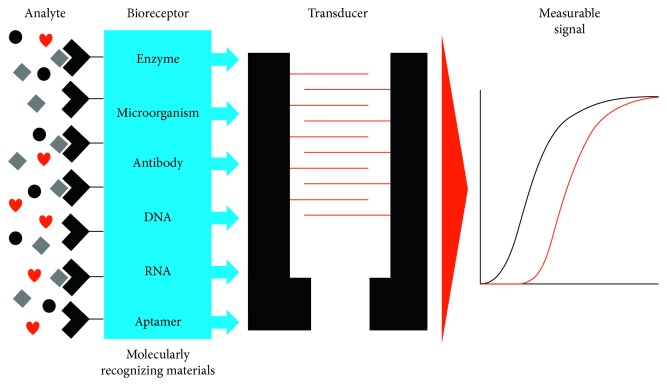
Basic principle of the biosensor. Three major portions including analyte, bioreceptor, transducer and measuring system are displayed.

**Figure 5:**
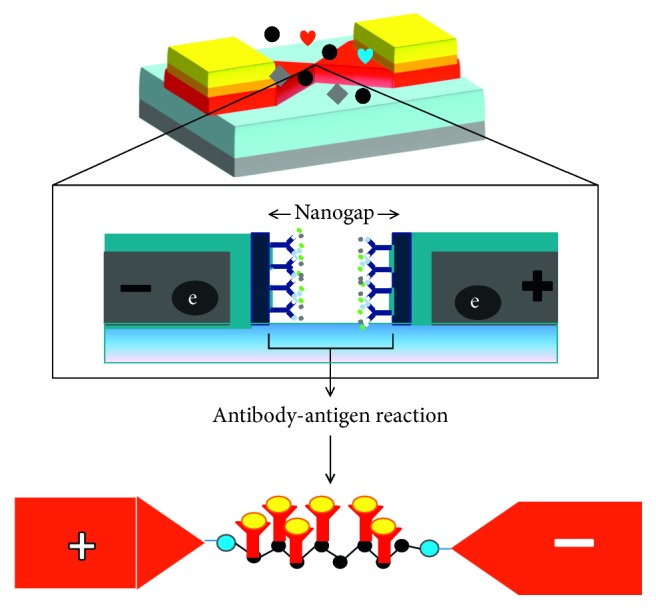
A label-free sensor. Interaction of antibody-antigen is shown using the dielectric electrochemical sensor.

**Figure 6:**
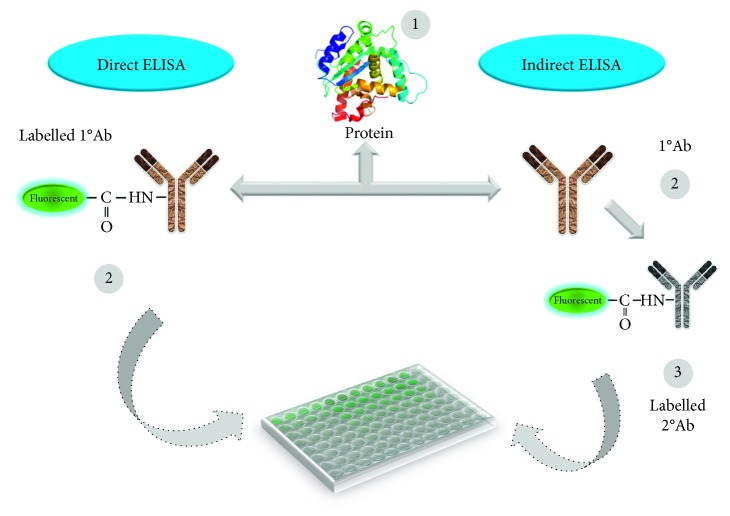
Enzyme-linked immunosorbent assay. Both direct and indirect methods are shown.

**Figure 7:**
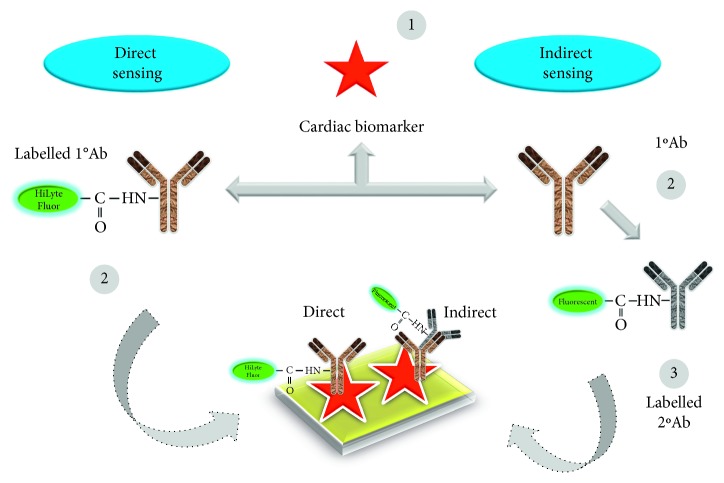
Direct and indirect identification methods of cardiac biomarkers by biosensing.

Using the above biomarkers, several sensing strategies have been generated in the past for cardiac diseases [25–28], based on the labelling and label-free strategies (Figure 3).  Table 1 summarizes the detection strategies using different cardiovascular biomarkers against the appropriate probe molecules. These sensing systems are mainly operating based on the transducer as stated elsewhere, in which the probe (receptor) molecule has been immobilized on the sensing surface to interact the analyte in the sample(s) to be analyzed. The transducer will convey the binding events, and it can be interpreted by the signal output (Figure 4) [37–43]. For detecting the cardiac biomarkers, the similar strategies have been followed and well demonstrated [25–28]. These biosensing systems can be used to survey the cardiovascular risks during noncardiac surgery. Different biosensors including surface plasmon resonance, electrochemical sensor, polymerase chain reaction, enzyme-linked immunosorbent assay, colorimetric analysis, and RAMAN spectroscopy were used to quantify the biomarker for cardiac diseases. Along with the sensing system, the appropriate probe for cardiac biomarker is also playing a vital role for early identification. In general antibody, DNA, RNA, and aptamer have been used as the probe to identify the cardiac biomarkers. These probes are more prevalent in the label-free methods such as surface plasmon resonance and dielectric sensors (Figure 5). On the contrary, antibody is also used in the gold-standard labelling enzyme-linked immunosorbent assay (ELISA) for detecting the clinical cardiac biomarkers [44]. There are two types of ELISA, namely, direct and indirect ELISA, which can be referred to detecting the cardiac biomarkers; furthermore, it is also suitable for the conventional sensing surfaces (Figures 6 and 7). Brain natriuretic peptide (BNP) is the neurohormone, widely adopted serological biomarker for analyzing the heart failure. It was proved that a high BNP level is usually found in patients with congestive heart failure. Identifying and quantifying the level of BNP in the blood are mandatory to diagnose the acute heart failure. N-terminal pro-B-type natriuretic peptide (NT-proBNP) is also one of the potential biomarkers for predicting heart failure. Magnetic bead-conjugated BNP with DNA aptamer-based sandwich strategy was used to detect BNP by electrochemiluminescence [45]. A researcher used two aptamers selected against BNP as a capture and reporter to quantify the level of BNP. In addition, it has been found that the elevated troponin T and troponin I have a significant correlation with cardiac injury. The troponin level in the normal blood is lower; after the onset of myocardial infarction, the level of troponin I is substantially increasing and is possible to measure in blood serum within four to six hours, and the peak concentration of troponin was found in 12 to 24 hrs after myocardial infarction, this will help diagnose the infarction. Detection of troponin at the lower level is mandatory to detect the myocardial infarction at an earlier stage and helps for further treatment. Troponin I was detected on the graphene oxide sheet by the fluorescence quenching method; the 5′-6-FAM-modified troponin aptamer was mixed with different concentrations of troponin; the fluorescence quenching and recovery of the solution were measured at 480 nm, and the detection limit was found as 0.07 ng/mL [46]. Apart from this method various direct- and indirect-sensing methods with different sensors have been used to identify the cardiac biomarkers for the perioperative period in noncardiac surgery patients.

## 6. Cardiac Risk Index

In the past, various cardiac risk indices have been followed and revised the cardiac risk index analysis by the following six variables to check the risk factors of the patients [47]. These include the history of heart failure, ischemic heart disease, stroke, preoperative insulin treatment, transient ischemic attack, and preoperative serum creatinine values (>152.5 mmol/l). The risk factors and the evaluation methods are summarized in Table 2 [47, 48].

## 7. Guidelines and Recommendations

There were guidelines and recommendations for patients undergoing noncardiac surgery. The Canadian Cardiovascular Society provides the following eight recommendations: (1) measure the level of N-terminal fragment of pro-BNP or brain natriuretic peptides before the surgery of the patients at the age above 65 years and ages from 45 to 64 years with a cardiovascular disease; (2) to enhance the estimation of perioperative cardiac risk, cardiopulmonary exercise testing or coronary computed tomography angiography or radionuclide imaging is need to be performed; (3) to be against the continuation or initiation of acetylsalicylic acid in order to prevent the perioperative cardiac event; (4) prior to 24 hrs of surgery, analyze *β*-blocker initiation or against *α*_2_ agonist; (5) maintain the angiotensin-converting enzyme inhibitor and angiotensin II receptor blocker 24 hrs before the surgery being started; (6) mandatory stop with the smoking habit before the surgery to be performed; (7) monitor the daily troponin level for 48–72 hrs after the surgery was carried out in the patients having confirmed higher level of NT-proBNP/BNP before the surgery, especially in the patient who has a Revised Cardiac Risk Index score equal to 1, aged between 45 and 64 years with an apparent cardiovascular disease, or aged 65 years and above; and (8) preparing for a long-term acetylsalicylic acid and statin therapy in patients suffering from myocardial infarction after the surgery [49].

## 8. Monitoring Perioperative Cardiac Risk with Computed Angiography

Patients with the advanced stages of coronary artery disease during the surgery have increased risk of cardiovascular events [50]. Continuous monitoring is necessary to avoid the risk factors. Coronary computed tomography angiography has been used to evaluate the patients prior to noncardiac surgery. It is a noninvasive well-established technique, which is effectively used to identify the left main and multivessel coronary artery diseases.

## 9. Conclusion

Every year, ∼50 million surgical operations have been performed in the United States; among them, 1.4 to 3.9% are complicated by a cardiac event. Accurate identification of risk factors is mandatory to reduce the cardiovascular risk especially in the patients aged above 50 years and having the history of cardiac problems. In this review, we discussed the possible cardiac risk factors and the key guidelines during the period of perioperation in the noncardiac surgery patients, and the efficient biomarkers for the cardiac disease diagnosis are discussed. The gleaned information here would help minimize the death rate during the perioperative period of the noncardiac surgery cases. It is important to notice that early identification of cardiac diseases with the suitable biomarkers is mandatory to avoid cardiac risks during the perioperative period.

## Figures and Tables

**Table 1:** Biomarker-associated measurements, risk factors, and guidelines.

Biomarker	Risks	Measurement	Probe	Limit of detection	Advantage/disadvantage	Clinical guide	Reference
BNP^*∗*^	Decrease in blood pressure	Immunofluorescent	Antibody	400 pg/L	Comparatively less sensitive	Monitor carefully with heart surgery patients	[29]
NT-proBNP	Decrease in blood pressure	Immunofluorescent	Antibody	10 ng/L	Good marker for surgery patients	Monitor carefully with heart surgery patients	[29]
ProBNP	Decrease in blood pressure	Immunofluorescent	Antibody	3 ng/L	Good marker for surgery patients	Monitor carefully with heart surgery patients	[29]
Troponin I	Heart attack	Electrochemical	Aptamer	30 pg/mL	Standard biomarker	Treatment for cardiac muscle damage	[30]
Troponin T	Contraction of skeletal and heart muscle and myocardial injury	Electrochemical	Antibody	1 pg/mL	Standard biomarker	Treatment for cardiac muscle damage	[31]
C-reactive protein	Inflammation in the arteries of the heart.	SPR^*∗∗*^	Antibody	10 pg/mL	Best target to predict the mortality with other markers.	Controlled diet and cholesterol level	[32]
Troponin I	Heart attack	Electrochemical	Antibody	1 pg/mL	Standard biomarker	Treatment for cardiac muscle damage	[31]
C-reactive protein	Inflammation in the arteries of the heart	SPR	Aptamer	10 pM	Comparatively less sensitive	Controlled diet and cholesterol level	[33]
C-reactive protein	Inflammation in the arteries of the heart	Voltammetry	Antibody	10 fM	High-sensitive. Biomarker for perioperative cardiovascular risk	Controlled diet and cholesterol level	[34]
Lactate dehydrogenase	Tissue damage	Amperometric	Aptamer and antibody	1 *μ*M	High specificity due to the aptamer	Treatment for enzyme regulation	[35]
High sensitivity troponin	Future heart attack	Electron mobility transistor	Aptamer and antibody	6 pg/mL	High specificity due to the aptamer	Treatment for cardiac muscle damage	[36]
BNP	Decrease in blood pressure	Electrochemical	Antibody	1 pg/mL	Good marker for surgery patients	Monitor carefully with heart surgery patients	[31]

^*∗*^B-type natriuretic peptide (BNP); ^*∗∗*^surface plasmon resonance (SPR).

**Table 2:** Analyses of cardiovascular risk index: comparison of National Surgical Quality Improvement Program and the Revised Cardiac Risk Index.

Criteria	National Surgical Quality Improvement Program	Revised Cardiac Risk Index
Interpretation	Elevated risk: 2 risk factors: 7% risk; ≥3 factors: 11% risk. Low risk: 0 risk factors: 0.4% risk; 1 risk factor: 0.9% risk	Refer a percent risk from web-based calculator (http://www.qxmd.com/calculate/calculator_245/gupta-perioperative-cardiacrisk)

Factors used	History of ischemic heart disease, cerebrovascular disease, heart disease, serum creatinine level, diabetes level, condition of undergoing intrathoracic surgery	Serum creatinine ≥1.5 mg/dL; age; surgery type

Validation and derivation	Prospective cohort; single hospital	Historical national database; multicenter

Advantages of screening	Used for more than a decade	Surgery-specific

Disadvantages of screening	Functional capacity is not a variable; advanced procedures such as laparoscopy were not used; only 0.2% of patients had severe aortic stenosis	Aortic stenosis and coronary artery disease are not variables; due to the elevation of unknown significance, possibility of over diagnosing myocardial infarctions

## Data Availability

All the data are fully available without restriction.

## Abbreviations

CK-MB: Creatine kinase MB
CK: Creatine kinase
AST: Aspartate aminotransferase
HDBH: Hydroxyl butyrate dehydrogenase
LDH: Lactate dehydrogenase
TnT: Troponin T
TnI: Troponin I
CRP: C-reactive protein
hsTnT: High-sensitive troponin
hFABP: Heart-type fatty acid-binding protein
miRNA: microRNA
MR-PAMAP: Midregional fragment of proadrenomedullin
ELISA: Enzyme-linked immunosorbent assay
BNP: Brain natriuretic peptide
NT-proBNP: N-terminal pro-B-type natriuretic peptide.
